# Policies and Programs for the Prevention and Control of Breast Cancer in Mexican and Latin American Women: Protocol for a Scoping Review

**DOI:** 10.2196/12624

**Published:** 2019-06-12

**Authors:** Igor Martin Ramos Herrera

**Affiliations:** 1 Center for Research on Health Information Systems and Management Department of Public Health University of Guadalajara Guadalajara Mexico

**Keywords:** breast neoplasms, public policies, primary health care, systematic review

## Abstract

**Background:**

Breast cancer has become a major public health problem around the world, especially in Mexico and Latin America. Screening for breast cancer, which involves self-examination, mammography, and clinical breast examination, is crucial for early diagnosis, which in turn is associated with improved outcomes and survival rates. Although breast cancer prevention and control activities are being implemented in Mexico and Latin America, as in many other countries, there are no comprehensive public reports that provide information on the number, type, and scope of these activities; the impact of the programs and actions implemented; and the policies that form the basis of these programs.

**Objective:**

This study aims to present the design of a protocol for a scoping review on the policies and action programs for breast cancer care in Mexico and Latin America, as well as their objectives and implementation plans.

**Methods:**

This scoping review is guided by the methodological reference framework proposed by Arksey and O’Malley. A systematic search of the following electronic databases will be performed: MEDLINE (PubMed), MEDLINE (EBSCOHost), CINAHL (EBSCOHost), Academic Search Complete (EBSCOHost), ERIC, ISI Web of Science (Science Citation Index) in English and Cochrane and MEDES-MEDicina in Spanish. A search will be conducted to identify relevant studies published between 2000 and 2018. Data will be analyzed and presented in descriptive statistics and qualitative content analyses with analysis matrices and semantic networks. The selected studies will be arranged according to the Specific Action Program, Prevention and Control of Female Cancer 2013-2018.

**Results:**

The intention is to perform this review during the first and second quarters of 2019 and present the results to health authorities by the first quarter of 2020. Results will also be sent for publication to an indexed journal by the second quarter of 2020.

**Conclusions:**

We present a protocol for a scoping review–type literature revision based on the Arksey and O’Malley methodology to be performed during the first quarter of 2019. According to this 6-stage methodology, we will identify the scientific publications that present or analyze first-level action policies and programs for breast cancer care in Mexican women, as well as the results of these policies and programs, if any. The outcome of this review will be used to define the basis of a research project intended to design an educational intervention strategy for the general public in Mexico to enable them to deal with this public health problem.

**International Registered Report Identifier (IRRID):**

PRR1-10.2196/12624

## Introduction

### Background

Breast cancer has become a major public health problem all around the world, especially in Latin America. In 2015, Mexico reported an incidence rate of 14.8 cases per 100,000 women [1], and the mortality rate was 15 cases per 100,000 women aged 20 years and older. Though self-examination and mammograms were recommended in 2007 as the best ways to diagnose this type of cancer [2], the Canadian Task Force on Preventive Health Care obtained recent evidence that self-examination does not reduce breast cancer mortality compared with those who did not practice it [3]; thus, health education is extremely important so that women can learn about this disease and feel encouraged to attend their medical service immediately after identifying any anomaly in their breasts, as advised by the Mexican Official Norm for Breast Cancer Detection and Control for each age group [4]. As in most of the countries of Latin America, breast cancer prevention and control activities are also being implemented in Mexico [5], but there are no comprehensive public reports that could provide information on the number, type, and scope of these activities; the impact of the programs and actions implemented; and the policies that form the basis of these programs.

One approach to gather this information is by conducting systematic reviews of scientific literature. There are different types of reviews, but in this study, we will focus on the scoping review or systematic exploratory review [6], as it is known in Spanish. Although designing a formal implementation protocol before carrying out a scoping review is not considered a requirement because of the possibility of study duplication [7], at an international level, there has been a trend toward publishing these protocols in scientific journals to shed light on the review methodology and scope.

### Theoretical and Evidence Base

Although protocols for scoping reviews regarding the prevention and control of chronic diseases such as obesity [8], breast cancer [9], or physical activity [10] have been published, at present, there is no reference in Mexico or Latin America of a scoping review about the policies and programs for the prevention and control of chronic diseases, including breast cancer. Therefore, a protocol was designed to perform a scoping review of the scientific publications that can identify the nature, extent, and range of breast cancer prevention and control policies and programs for Mexican and Latin American women to evaluate how both align with the existing actions on an international level and identify the gaps in this regard.

The Official Mexican Standard NOM-041-SSA2-2011 [4] recognizes that the main risk factors for this disease are grouped in 4 categories: (1) biological risk, including gender, age, inheritance, history of breast changes, extended menstrual cycle, and high breast density; (2) iatrogenic or environmental risk, including exposure to ionizing radiation and thorax radiation therapy; (3) risk in the reproductive history, including pregnancy absence, first pregnancy at an advanced age, and perimenopausal and postmenopausal hormone therapy lasting more than 5 years; and (4) high-risk lifestyles, including high-carbohydrate and low-fiber diets, high-fat diet, obesity, physical inactivity, alcohol consumption of more than 15 g/day, and smoking. Early diagnosis is one of the most important elements for successful treatment, and its delay means that patients are diagnosed at advanced cancer stages [11-13], which results in poor prognosis and survival rates.

Current health policies regarding breast cancer, which are included in the official standards, state decrees, and clinical practice guidelines, are based on primary and secondary prevention [4,5]. These levels of prevention focus on minimizing lifestyle risks, but they also focus on early diagnosis and timely care, which leads to improved survival rate for those women who receive a timely breast cancer diagnosis.

The Specific Action Program, Prevention and Control of Female Cancer 2013-2018 [5] identifies breast cancer as a public health problem. This action program issues from the Sectorial Health Program, which derives from the National Development Plan in Mexico 2013-2018 [14]. The specific action program states the objectives and strategies that shall be followed by health authorities to (1) increase the joint responsibility of men and women in the prevention and early detection of cervical-uterine and breast cancer; (2) reinforce the detection, follow-up, and timely quality treatment of cervical and breast cancer cases, and (3) contribute to the convergence of cancer information systems between the institutions of the National Health System.

Prevention for breast cancer in women can be primary or secondary. Primary prevention includes all the activities that are aimed to reduce the onset of breast cancer by controlling the causal factors and the predisposing or determining factors [2,15], with the aim of reducing the incidence of this disease. According to the Official Mexican Standard 041-SSA2-2011 [4], primary breast cancer prevention actions include good health promotion, specific protection, and chemoprophylaxis.

Secondary breast cancer prevention, on the other hand, is completely oriented toward early disease diagnosis in an incipient stage (without clinical manifestations), which means looking for signs and symptoms of the disease in women who *appear to be healthy* [2]. The same Official Mexican Standard 041-SSA2-2011 identifies early diagnosis and timely treatment programs to limit the damage that cancer may cause, which can be achieved through periodic medical examination and planned case search. This official standard establishes that prevention activities, in general, include educating the population about the risk factors, promoting healthy lifestyles that may contribute to reducing breast cancer morbidity, as well as encouraging the demand of early detection to improve the opportunity of diagnosis and treatment.

Finally, breast cancer detection activities consist of 3 types of specific interventions that address the female population according to their age and vulnerability group: (1) self-examination to identify initial symptoms, (2) clinical examination for early identification, and (3) mammograms for preclinical phase identification. Therefore, educational actions at a preventive level must focus on orienting and educating women so that they perform these 3 types of intervention routinely from the age of 20 years onward and with the frequency established by the standards.

### Objective

On this basis, in the words of Arksey and O’Malley [16], a scoping review would help to describe in more detail the actual primary and secondary prevention programs for breast cancer that have been implemented by the countries in the Americas, thereby providing a summary of those findings to policy makers, health authorities, and other researchers. Therefore, the objective of this study is to present the design of a scoping review protocol on the policies and action programs for breast cancer care in Mexico and Latin America, as well as their objectives and implementation plans.

## Methods

### Design

This scoping review protocol was based on the methodological framework proposed by Arksey and O’Malley [16], which was later modified by the Joanna Briggs Institute [17]. Though this work is based on Arksey and O’Malley’s guidelines, other guidelines for systematic reviews were also examined, such as the Preferred Reporting Items for Systematic Review and Meta-Analysis (PRISMA) protocols [18] or the Consolidated Standards of Reporting Trials [19], but these were intended for reviews on clinical evidence and not for primary health care programs or scoping reviews. However, we performed a checklist using the PRISMA Extension for Scoping Reviews (PRISMA-ScR) [20] guidelines.

A systematic search will be performed of the main electronic databases available internationally and that can be accessed in full text through the Digital Library of the University of Guadalajara [21], Mexico. The databases that will be consulted are as follows: MEDLINE (PubMed), MEDLINE (EBSCOHost), CINAHL (EBSCOHost), Academic Search Complete (EBSCOHost), ERIC, ISI Web of Science (Science Citation Index) in English, Cochrane, and MEDES-MEDicina in Spanish. The searching period will be between 2000 and 2018. The protocol will be based on the 6 stages established by the Arksey and O’Malley’s [16] methodology.

Some adaptations to these stages will be made as the process advances, with the intention of ensuring a feasible approach, consistent with the existing literature. Each one of these stages is described as follows.

### Stage 1: Identifying Research Questions

According to the guidelines of the methodological framework, an initial iterative process of revisions must be carried out to raise 1 or more questions that may guide the research. For this purpose, the iterative process has already begun with the intention of identifying existing parameters that can give structure to the review as well as define in operational terms, database consultations about breast cancer diagnosis within the framework of the breast cancer prevention and control actions performed by the health authorities in Mexico and Latin America. This initial search yielded very few results, but among those, we found the Specific Action Program, Prevention and Control of Female Cancer 2013-2018 [5], whose guidelines we decided to take as an important preliminary finding to raise the base questions for the review because we consider that they reflect real actions that are developing in the region. Textbox 1 shows the questions proposed for this scoping review following such guidelines, as well as the definitions that will be used to make this search operational.

**Research questions and their operational definitions (modified from the Specific Action Program, Prevention and Control of Female Cancer 2013-2018 [5]).**

Which breast cancer prevention and control policies in Mexico and Latin America have been analyzed in the national and international scientific literature?

- Guaranteeing effective access to quality health services
- Improving the breast cancer detection and care process
- Establishing breast cancer risk communication strategies
- Focusing on breast cancer prevention and detection actions
- Developing and spreading performance evaluations of breast cancer screening programs

Which is the nature (type), extension, and range of these policies and/or programs according to those reports?

- Breast cancer prevention and control strategies: best practices in line with the sociodemographic characteristics of the populations to improve the access, coverage, and quality of the actions that promote health, detection, diagnosis, and follow-up of female cancer
- Implementation based on scientific evidence of national and international experiences and with gender perspective
- Interinstitutional coordination to universalize procedures, practices, efforts, and impacts; among those, the efforts to universalize breast cancer screening through mammography
- Participation of the organized civil society and the citizens in processes that improve access to services and politically influenced actions (citizen monitoring and oversight)
- Reducing health gaps, according to the epidemiological trends in female cancer and the sociodemographic characteristics of the populations
- Health service expense, as a responsible investment, regarding the sociodemographic characteristics of the populations
- Systematic monitoring and evaluation to continuously improve the program
- Coordinating together with the institutions of the National Health System to universalize an information registry and statistical sources with an ethnic focus and gender perspective to improve epidemiological vigilance.

Which is the existing frame of reference for female breast cancer prevention and control policies and/or programs on a national and international level?

- International Breast Cancer Prevention and Control Plans
- National Development Plans
- Sectorial Health Programs

### Stage 2: Identifying Relevant Studies

Even though the objective of a scoping review–like revision work is to account for the research questions in the broadest sense, establishing parameters that guide the search strategy is necessary. By this logic, inclusion and exclusion criteria for the eligibility of the studies, the databases to be used and the key consultation words or terms were determined.

#### Eligibility Criteria

On the basis of the same methodology, eligibility criteria must be established for identified studies about breast cancer prevention and control policies and programs. Thus, the studies that will be selected in this review must comply with the following inclusion criteria:

1. Studies about public policies or programs regarding breast cancer prevention and control published between January 2000 and December 2018.
2. Studies published in English and Spanish preferably.
3. Works on public policies or programs applicable to female human subjects of any age group.
4. Review studies that include systematic revisions, meta-analysis, meta-synthesis, other scoping reviews, and gray literature (annual, research, technical, or project reports; working papers, government documents, white papers and evaluations, etc).

Likewise, the following exclusion criteria shall apply:

1. Studies about public policies or programs addressing any other type of cancer.
2. Profit-seeking advertising documents.

#### Databases

Databases to be consulted are as follows: MEDLINE (PubMed), MEDLINE (EBSCOHost), CINAHL (EBSCOHost), Academic Search Complete (EBSCOHost), ISI Web of Science (Science Citation Index), and Scopus in English; and SciELO, Cochrane, and MEDES-MEDicina in Spanish. The author considers that these databases are the ones that most probably contain the range and scope of the studies included in this review.

#### Search Strategy

In the indicated electronic databases, an initial search about breast cancer prevention and control policies and programs will be performed by 2 researchers. The following filter shall be applied to the initial search: Mexico or Latin America. By Latin America, we will include documents from the all the countries in Central, South America, and the Caribbean.

The search terms will be defined based on the initial database consultations; these words will include the Medical Subject Headings terms for the database search in English and the terms from Health Sciences Descriptors of the Virtual Health Library of the Pan American Health Organization for the database search in Spanish. The search terms shall include the words “policies,” “public policies,” “programs,” “strategies,” “laws,” “prevention,” and “control” combined with “breast cancer” and “malignant neoplasms,” both in English and Spanish languages.

Thereafter, a manual search of relevant documents that have not been enlisted in the electronic databases search will be implemented. Furthermore, literature will be searched in relevant sites such as the Pan American Health Organization and the World Health Organization, health departments in Mexico and Latin American countries, Academic Google, and abstract databases of specialized conferences and meetings. Finally, we will search for gray literature in the sites provided by the previous consultations. All references shall be handled through a bibliographic citation management software to organize references and eliminate duplicates.

### Stage 3: Study Selection

Documents containing information about breast cancer prevention and control policies and programs are of interest to this review. Therefore, Mexican and Latin America’s public policies and health education campaigns, healthy lifestyle promotion, timely detection, and identification of environmental and genetic factors shall be included in this analysis. To this end, once we obtain the results from the previous stage, the titles, overviews, and executive summaries shall be reviewed to identify topic-relevant literature, which will be checked per the inclusion and exclusion criteria to verify their eligibility. The documents complying with the inclusion criteria shall be taken into account for this review. To this end, 2 assistant reviewers will select a first round of documents; afterwards, each one shall submit their work for consideration to the other reviewer. In case a discrepancy should arise among them, a third reviewer will be consulted to solve the differences in their criteria. The work will be done under constant supervision to ensure high standards during the scoping review.

### Stage 4: Data Representation

The documents and reports selected in the previous step will be submitted to the data extraction process through specifically designed forms. Resulting data will be first analyzed with descriptive statistics and then through a qualitative content analysis, thus ensuring that the key elements of this search are reflected appropriately. For this, the 2 main reviewers will extract data independently, and the process of comparison will be performed thereafter.

Data extracted from the documents shall include the following: (1) authors, (2) year of publication, (3) document source (public or private), (4) type of document (scientific, political, gray literature, etc), (5) targeted population, (6) subject (public policy or action program), (7) proposed action or activity, (8) level of application (federal, state, municipal, or local), (9) field of action (primary, secondary, or tertiary prevention), and (10) political emphasis (primary attention, treatment, or survival). The data format could be modified as the review progresses and as we become familiar with the data found in the documents.

### Stage 5: Result Classification, Synthesis, and Report

Finally, the documents will be arranged according to the Specific Action Program, Prevention and Control of Female Cancer 2013-2018 [5] in a manner such that they can be categorized as shown in Textbox 1 and the way in which each applicable policy or program—either in a geographic, economic, infrastructure or breast cancer detection process sphere—can be identified.

As no primary source data will be obtained, the approval of an ethics or research committee is not necessary. Finally, quantitative data will be reviewed and presented as descriptive statistics and qualitative data as analysis matrices and semantic networks. Stage 6, the consultation exercise, will be considered once stage 5 is finished.

## Results

The report of this scoping review will be verified using the PRISMA-ScR Checklist for scoping review reports [20]. The intention is to perform this review during the first and second quarters of 2019. Results will also be sent for publication by the second quarter of 2020 to an indexed journal. The final aim is to present the results of this review to local and national health authorities by the first quarter of 2020, as well as present them in different scientific events and national conferences and meetings.

## Discussion

We present a protocol for a scoping review–type literature revision based on the Arksey and O’Malley’s [16] methodology during the first and second quarters of 2019. According to this 6-stage methodology, we will identify the scientific publications that present or analyze first-level action policies and programs for breast cancer attention in Mexican and Latin American women, as well as their results. The outcome of this review will be used to identify and define the basis of a research project intended to design an educational intervention strategy for the general public in Mexico, to contribute toward raising the awareness for breast cancer and its prevention. Likewise, it attempts to identify the gaps that still exist in public health policies to contribute to the development of well-structured and well-financed comprehensive care programs [22,23].

## Abbreviations

PRISMA: Preferred Reporting Items for Systematic Review and Meta-Analysis
PRISMA-ScR: Preferred Reporting Items for Systematic Review and Meta-Analysis Extension for Scoping Reviews
